# The effect of playing e-sports games on young people’s desire to engage in physical activity: Mediating effects of social presence perception and virtual sports experience

**DOI:** 10.1371/journal.pone.0288608

**Published:** 2023-07-27

**Authors:** Wang Ningning, Cheng Wenguang

**Affiliations:** 1 School of Physical Education, Liaoning Normal University, Dalian, 116029, China; 2 Graduate Students’ Affairs Department, Shenyang Sport University, Shenyang, 110102, China; 3 School of Management and Journalism, Shenyang Sport University, Shenyang, 110102, China; Universidad Central de Chile, CHILE

## Abstract

E-sports game experiences can help enhance young peoples’ willingness to participate in sports and fitness. However, e-sports game studies have mostly focused on users’ violent tendencies and aggressive behaviors, and less attention has been paid to the positive effects on young peoples’ sports health. The purpose of this study is to provide reasonable guidance young people away from sedentary, addictive and other negative behaviors and to promote active sports and healthy exercise and development. Following the random sampling criteria, questionnaires were distributed to Chinese young people aged 14 to 24 through gaming communities as well as social media platforms, and 1608 valid questionnaires were obtained after eliminating invalid ones. The influence mechanism of e-sports game experience on young peoples’ intention to participate in sports and fitness was examined empirically by using factor analysis and structural equation modeling. The e-gaming scenes (β = 0.399, p<0.01), virtual sports experience (β = 0.257, p<0.01), and social presence (β = 0.258 p<0.01) each had a significant positive effect on young peoples’ intention to participate in sports and fitness; virtual sports experience [OR]0.099, 95%: CI 0.077–0.121 and social presence[OR]0.052, 95%: CI 0.035–0.071, were known to have mediating utility in the e-gaming scenario’s influence on young peoples’ intention to participate in sports and fitness. Using the significant features of sports video games such as entertainment and simulation to awaken the interest and willingness to participate in young people sports is an innovative way to accelerate the development of mass sports.

## 1. Introduction

The Hangzhou Asian Games in 2022 will include e-sports in the official sports competition, which has been developing rapidly worldwide. E-sports games are physical and intellectual rivalries between people with the help of various hardware and software at the core of modern technology and the environment created by it, which is similar to the equipment and venues in traditional sports. In recent years, China’s video game industry has developed rapidly, and according to the 2021 Game Industry Annual Report, the actual sales revenue of China’s game market is 296.513 billion yuan, an increase of 17.826 billion yuan over the previous year, with a year-on-year growth of 6.4%. At the same time, the number of Chinese game users is expected to reach 666 million, with young people under the age of 25 accounting for approximately 30% of this group, providing a solid force for the development of the e-sports game industry. It is clear that the economic benefits of the video game industry are becoming more and more prominent and have gradually become the focus of attention from all walks of life [1]. At the same time, e-sports originated from the differentiation of the sports attributes of video games, and there are many intersections between e-Sports and many fields such as culture, sports, and games, reflecting the basic needs of modern human society for the pursuit of excellence in game competition in terms of instinct, desire, and progress [2,3]. It is evident that the new idea of sports health promotion through sports video games that enables young people to explore and integrate innovations in their gaming behaviors and also enhance sports health is gradually gaining full academic recognition [4,5]. However, the current unhealthy behaviors such as game addiction, visual health problems, and sedentary behavior among young people are the causes of the negative effects such as low real participation rate and low willingness to participate in physical activity among the general population, as well as the urgent need to address the development of young people health.

The social cognitive theory proposed by Bandura, which emphasizes the role of individual factors in hands-on learning and considers the shaping of behavior by behavioral outcomes as an automatic process, provides theoretical support for the shift of young people sports video games to participation practices. Reviewing the previous studies, most of the studies on sports video games in the early 21st century focused on users’ tendency to violence, aggressive behavior [6], and game addiction [7] and other negative issues. With the gradual enrichment of video game genres and increasing audiences, the positive functions of sports video games have gradually attracted the attention of scholars at home and abroad. Enhancing the learning of technical sports skills through virtual reality technology has the ability to induce positive emotions, reduce negative emotions, and reduce state anxiety in gamers [8]. Virtual reality (VR) systems have greater potential for training technical behaviors and have attracted significant attention in the field of sports, such as learning basic skill structures in VR and transferring them to the real world [9]. Lopez (2021) by constructing a media practice model, we found that the features of sports video games, such as entertainment and simulation, are very effective in enhancing users’ positive cognitive tendencies and emotional states towards sports [10], and can significantly increase users’ interest in participating in sports and their willingness to participate in realistic sports [11] Nobuko, Ballard, and other foreign scholars found that sports video games can effectively improve the responsiveness, teamwork, coordination, self-control, and social presence perception of young people [12]. Among them, the social presence perception behavior is always present during the user’s experience of sports video games [13]. According to Bandura’s cognitive learning theory, learning has an important role in influencing the formation of individual attitudes and behavior change [14]. In the existing studies, some scholars have demonstrated the real existence of young peoples’ social presence perceptual behavior within the game by setting up different game scenarios and speculated that this behavior may trigger young peoples’ motivation to participate in realistic sports-related activities, such as enhancing the number of discussions between users and peers or increasing the frequency of practice [15,16]. However, a glance at the available studies reveals a more fragmented effect regarding users’ social presence and perceptual behavior within sports video games on their willingness to participate in reality. At the same time, changes in the degree of perception are a core indicator of the effectiveness of individual learning behaviors and one of the important antecedent variables influencing individual motivation, mood, attitude, and behavior change [17]. Some scholars have pointed out that the increased degree of virtual sports experience of sports video game users can stimulate their positive cognitive tendencies and emotional states towards real sports [18,19], but there have been few studies. In-depth studies have been conducted to explore the intrinsic relationship between virtual sports experiences and users’ intention to participate in sports and fitness.

In view of this, this paper intends to investigate the intrinsic mechanism of the gaming scene, social presence perception, and virtual sports experience on young people sports and fitness participation intentions from sports video games and construct a path model of the influence of the gaming scene on young people sports and fitness participation intentions, so as to provide a reference for stimulating young people’s interest in realistic sports and fitness, awakening users’ willingness to participate in reality, and comprehensively implementing the national fitness strategy initiative.

## 2. Hypothesis of research and conceptual model

### 2.1. The direct effect of e-game scenarios on the willingness to participate in sports and fitness

Sports video games are video games with various types of electronic devices as the running medium and with the content of manipulating virtual game characters to participate in sports. The beautiful video game scenes and beautiful music usually make young people feel relaxed and happy during the game, and it is seen that the scenario features such as video game entertainment, novelty, simulation, interaction, and transferability attract a large number of young people to participate in the experience. And young peoples’ intelligent behavior in video games largely determines the development of the game process, dominates the fate of the game characters, and rewrites the final ending of the game. This high sense of manipulation and the uncertainty of the ending users’ deep involvement motivate player young people to explore unlimited possibilities in realistic sports. The willingness to participate in real sports refers to the subjective desire of young people to participate in sports fitness, sports competitions, and related sports consumption activities after experiencing sports video games [20] which reflects the dynamic changes in users’ psychological and behavioral levels from the initial exposure to the re-engagement process. Sports video games can be divided into two types, physical and non-physical games, depending on whether young people need to control the game characters through real body movements. Somatosensory sports video games, as new types of video games that control virtual characters through physical changes, can motivate young people to enjoy the fun of sports during the game experience [21]. Somatosensory games such as "Dance 2021", "Aerobic Boxing", and "Fitness Ring Adventure" highly integrate virtual experience with realistic participation, and their excellent simulation and reproduction enhance the fun of the game while positively influencing young people’s willingness to explore and engage with sports. Non-sensory sports video games are interactive games that rely on conventional electronic devices such as keyboards and handles. For example, in the NBA 2K series of basketball games, the sense of accomplishment and satisfaction obtained when young people manipulate players to run, break, layup, shoot, and score in the game will drive young people to imitate and demonstrate in reality [22]. Accordingly, the hypothesis is proposed.

H1: The e-gaming scene has a positive effect on the willingness to participate in sports and fitness.

### 2.2. The mediating effect of social presence perception on the gaming scene and the willingness to participate in sports and fitness

The concept of "presence" originated from "telepresence", proposed by Minsky in 1980 [23], and evolved through "virtual presence". The concept of "social presence" originated from Minsky’s "telepresence" in 1980 and evolved from "virtual presence". As a concept describing psychological perception, the dimensionality of social presence is applicable to the individual’s perceived situational state. At present, the dimensional division of social presence is divided into two categories: technical factors and social factors, among which, technical factors mainly refer to the characteristics of the network environment in which individuals are located; while social factors are mainly reflected in the emotional aspects, such as belongingness, intimacy, interactivity, authenticity, etc. It can be seen that the social proximity of young people in the e-sports game scenario mainly describes the psychological state in the virtual context and is a reflection of the real social elements that individuals are feeling in the virtual game scenario [24–26]. During sports e-Sports games, social presence is mainly reflected in the physiological, emotional, trait, and identity perceptions felt during the virtual game, reflecting the degree of similarity between the virtual self-presentation and the real self [27]. By manipulating the game character, the young people feels present with the avatar, and through repeated imitation, learning, and recording of sports game knowledge, skills, and thinking, he or she integrates the avatar into his or her body schema and makes plans for different gaming scenarios to achieve his or her psychological [28]. As a form of virtual self-presentation, the visual image and behavioral performance of young peoples’ avatars in different e-gaming scenarios are highly variable, and young peoples’ perception of the visual image of their avatars and the behavioral meaning of their avatars is a key factor influencing their participation in sports fitness and sports competition [29]. Studies have shown that the interactive nature of sports e-Sports game scenarios provides a potential environment for young peoples’ social presence perceptions and that young peoples’ social presence perceptions mediate the relationship between e-Sports game scenarios and sport and fitness participation intentions. Specifically, the hotness of the opening scene of e-sports games, the intensity of physical collisions, and the reversibility of player transfers give young people strong sensory stimulation and inject sports knowledge, skills, culture, and rules into the subconscious, driving young people to consistently apply sports game-related knowledge and skills to sports practice. When users in the process of playing encounter unfamiliar game rules or unfamiliar sports skills, they will tend to solve the problems they encounter in realistic scenarios, i.e., young peoples’ social presence perceptual behavior influences the willingness to participate in sports and fitness. On the other hand, according to embodied cognition theory, users who have some realistic sports foundation can better understand and assimilate knowledge. Young people continuously combine the data from heir own sports experiences and biographies into the game to complete the convergence of old and new knowledge, pique their interest in physical education learning and urge for active inquiry, and increase their willingness to engage in sports and fitness.Accordingly, the hypothesis is proposed.

H2: The e-gaming scene has a positive effect on social presence perception.

H3: Social presence perception has a positive effect on exercise and fitness participation intention.

H4: Social presence perception mediates the relationship between e-gaming scenarios and willingness to participate in sports and fitness.

### 2.3. The mediating effect of virtual sports experiences on the e-sports scene and the willingness to participate in sports and fitness

The development of mobile terminal technology and the vertical segmentation of the Internet market have provided the possibility of a virtual sports experience for young people. Virtual sports experience refers to the reactions, feelings, and ideas about human movement generated by individuals through participation, practice, or observation of various virtual sports processes in the context of the development of Internet information technology. Generally speaking, the ways of obtaining virtual sports experience are mainly divided into the following two aspects: first, the sports experience obtained by individuals participating in sports e-sports games, and second, the sports experience obtained by watching others’ sports e-sports games [30]. Research has shown that the degree of virtual sports experience, as a key factor influencing the intention to participate in sports and fitness, has a dominant effect on young people participation in real sports. Specifically, in real soccer, left-footed players subconsciously choose the superstar that matches their dominant foot when experiencing soccer video games, which leads to a sense of alternative accomplishment, and this alternative accomplishment motivates young people to think as players rather than just manipulating the game character for the game experience from the player’s perspective. At the same time, Jamie noted that this alternative sense of accomplishment leads to a deeper intrinsic connection with the real sports program, which affects young peoples’ motivation to participate in real sports [31,32]. Related studies have shown that the demonstration of professional sports technology, the realistic reproduction of sports scenarios, and the expression of sportsmanship within sports video games play an important driving role in enhancing young people virtual sports experiences and promoting real sports participation. Haichun, Peter noted that sports video games can significantly enhance the level of young people virtual sports experience and help young people in real sports fitness to build positive and active attitudes and values, promote young peoples’ active participation in sports and fitness; recreation, etc.; and develop regular exercise habits [33,34].

H5: e-gaming scenarios have a positive influence effect on virtual exercise experience.

H6: virtual sports experience has a positive influence on the intention to participate in sports and fitness.

H7: virtual sports experience acts as a bridge between e-gaming scenarios and willingness to engage in sports and fitness.

Related research has shown that social presence perception strategies can improve young peoples’ knowledge, skills, culture, and participation in sports programs [35]. Social presence perception is one of the most important ways for individuals to improve their knowledge and skills, as well as their willingness to behave, in both real-world and virtual environments.

H8: By combining hypotheses H2 and H6, it is hypothesized that social presence perception has a positive effect on virtual engine experience.

H9: Perceptions of social presence and virtual sports experience act as a bridge between e-gaming scenarios and willingness to participate in sports and fitness.

## 3. Study design

### 3.1. Selection and measurement of variables

The model summary includes four variables: e-gaming scene, social presence perception, virtual sports experience, and intention to participate in sports and fitness. The questions are taken from established scales, and all are measured using a 7-point Likert scale. The "gaming scene" dimension is based on the revised scales of Wu et al. (2010) [36], Ortiz de Gortari et al. (2015) [37], Muhterem D (2017) [38], and Bueno (2020) [39], and is based on the following scales: "entertainment, novelty, simulation, interaction, and transferability"; the social presence perception measure was borrowed from Tu et al. (2002), He A Z (2020) [40], and others’ scales, and was developed from "engagement, closeness, belongingness, immersion" The virtual exercise experience scale is based on Kim (2006) [41], Chen L (2018) [42], and measures "exercise scenario, immersion experience, embodied cognition, and emotional experience"; the intention to participate in exercise and fitness scale is based on Seo et al. (2008) [43], and Yi Z Q et al. (2021) [44], which measured "willingness to engage, willingness to participate, willingness to sustain, willingness to repeat, and willingness to prioritize". At the same time, to ensure that the scales are consistent with the research scenario of this paper, the selected items and the measurement environment are "online games", "virtual games," and "gaming behavior," etc. to ensure the validity and credibility of the model as a whole.

### 3.2. Sample structure and data collection

#### 3.2.1. Data collection

To ensure the objectivity and adequacy of the data, this study followed the criteria of random sampling, and the researcher used approximately 1,820 posts and responses published in the Xiaomi online brand community activity section and sports game community section, as well as the 3DMGAME forum, basketball NGA player community, and Steam player community, from August 2021 to February 2022. The data was collected. The respondents were young people aged 14 to 24, and 212 invalid questionnaires with less than 3 months of game seniority, less than 1 day of online game time per week, and less than 1 hour of game time each time were excluded, and 1608 valid questionnaires were obtained, with a questionnaire efficiency of 88.35%. In this study, the coding related to sports game experience refers to Tobón et al.’s classification method to develop a questionnaire on the influence of eSports game experience on young people’s intention to participate in sports and fitness [45]. Tobón et al. distinguish between game elements, which include explicit interface elements such as points, badges, and leaderboards, and game mechanics, which explain how game elements, rules, and user characteristics combine with each other and why they produce a game experience. This approach helps to provide continuous and immediate feedback to young people gamers, whose growth and engagement progress in the community can be practically felt through changes in game elements. This study was approved by the Ethics Committee of Liaoning Normal University (approval number: 2021027), and all participants (under 18 years old and over 18 years old) signed a written informed consent form.

#### 3.2.2. Sample organization

Among the 1608 valid questionnaires, 925 (57.52%) were male and 683 (42.48%) were female; the age distribution of the sample was 279 (17.35%) below 16 years old, 345 (21.46%) between 17 and 19 years old, 460 (28.61%) between 20 and 22 years old, and 524 (32.59%) between 23 and 24 years old. On the whole, the demographic characteristics of the sample were basically in line with the study expectations, and the questionnaire results were somewhat representative.

### 3.3. Evaluation of reliability

In order to avoid the interference of the scale with the Western cultural background and to ensure the accuracy, relevance, and validity of the scale items, two teachers from foreign language colleges of universities were invited to translate the foreign language scale items according to the Chinese language habits and grammatical structure and then discuss them with two experts in sports psychology, two experts in sports training, and two experts in sports sociology several times to control the content of the scale as a whole. The scale was modified and adjusted according to the purpose of the study. A small number of inappropriate items were eliminated, and the wording of the items was contextualized to ensure the reliability and validity of the scale.

For the reliability test, the internal consistency coefficient (Cronbach’s α) and the combined reliability (CR) were used to check the scale components. The results are shown in Table 1: the factor loadings of the four constructs were above 0.7, the Cronbach’s alpha coefficients were between 0.811 and 0.940, and the total scale Cronbach’s alpha value was 0.916, which was greater than the empirical value of 0.7; the combined reliability CR was between 0.855 and 0.931, which was greater than the empirical value of 0.5, and the p < 0.01, indicating that the scale has good reliability and internal consistency.

**Table 1:** Shows the factor loadings as well as the confidence validity analysis for each variable.

Variables	Measurement term	Factor loadings	Cronbach’s α	CR	AVE
E-gaming scenes	CJ1	0.851	0.940	0.931	0.730
	CJ2	0.851			
	CJ3	0.876			
	CJ4	0.841			
	CJ5	0.853			
Social Presence	LC1	0.741	0.811	0.855	0.596
	LC2	0.78			
	LC3	0.773			
	LC4	0.792			
Virtual Sports Experience	TY1	0.791	0.875	0.888	0.624
	TY2	0.785			
	TY3	0.778			
	TY4	0.785			
	TY5	0.779			
Willingness to exercise fitness	CY1	0.786	0.915	0.895	0.629
	CY2	0.797			
	CY3	0.797			
	CY4	0.793			
	CY5	0.793			

For the validity test, the scale was verified using the sphericity test (KMO and Bartlett’s) and the average variance extracted (AVE) values. The values of KMO and Bartlett’s were 0.936, which passed the empirical value criterion of 0.7 and P < 0.001. As shown in Table 1, the AVE values of each construct ranged from 0.596 to 0.730, which were greater than the empirical value criterion of 0.5 and passed the convergent validity test.

## 4. Study results

### 4.1. Methodological bias

Because the survey respondents self-completed the survey, a common method bias test is required before statistical analysis of the effect of e-gaming scenarios on young people willingness to participate in sports and fitness (Lu X al., 2020). The questionnaire in this study was controlled by using anonymous completion, reverse design of questions, and mixed ranking. The procedure was as follows: by constructing the validated factor analysis model M1 and the model M2 containing the method factors, the main fit indices of both were analyzed and the results were found to be: ΔCMIN/DF = 0.067, ΔCFI = 0.001, ΔGFI = 0.002, ΔIFI = 0.001, ΔNFI = 0.002, ΔRMSEA = 0.002, ΔSRMR = 0.001, where the amount of variation is less than 0.1 for CFI and TLI, and less than 0.05 for RMSEA and SRMR, indicating that there is no serious problem of common method bias among the variables in this study.

### 4.2. Description of variables and correlation analysis

The mean values of latent variables in this study ranged from 4.089 to 4.437, with small data volatility and a standard deviation <1.5. The fit between the variables was good, and the Pearson correlation coefficients between the four variables of e-gaming scene, virtual sports experience, social presence perception, and sports fitness participation intention were all positive, which initially confirmed the above hypotheses and satisfied the statistical requirements (Table 2).

**Table 2:** Variable description and correlation analysis results.

Correlation	M	SD	E-gaming scenes	Virtual Sports Experience	Social Presence	Willingness to exercise fitness
E-gaming scenes	3.495	0.888	1			
Virtual Sports Experience	2.878	0.489	0.280**	1		
Social Presence	4.100	0.606	0.348**	0.319**	1	
Willingness to exercise fitness	3.944	0.615	0.538**	0.413**	0.456**	1

** At the 0.01 level (two-tailed), the correlation is significant.

### 4.3. Goodness-of-fit test for models

**Fig 1:**
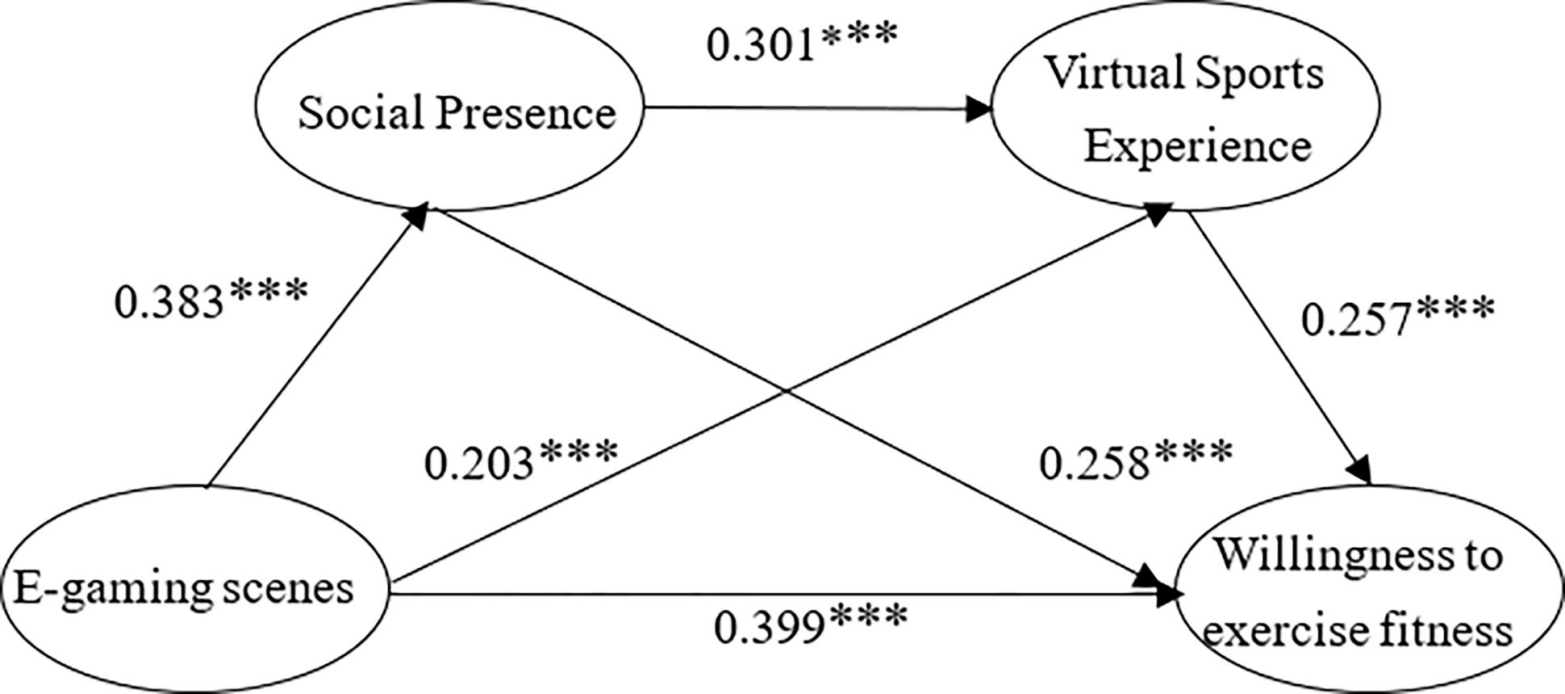
Structural model of the influence of e-sports game experience on young peoples’ willingness to participate in sports and fitness.

**Fig 2:**
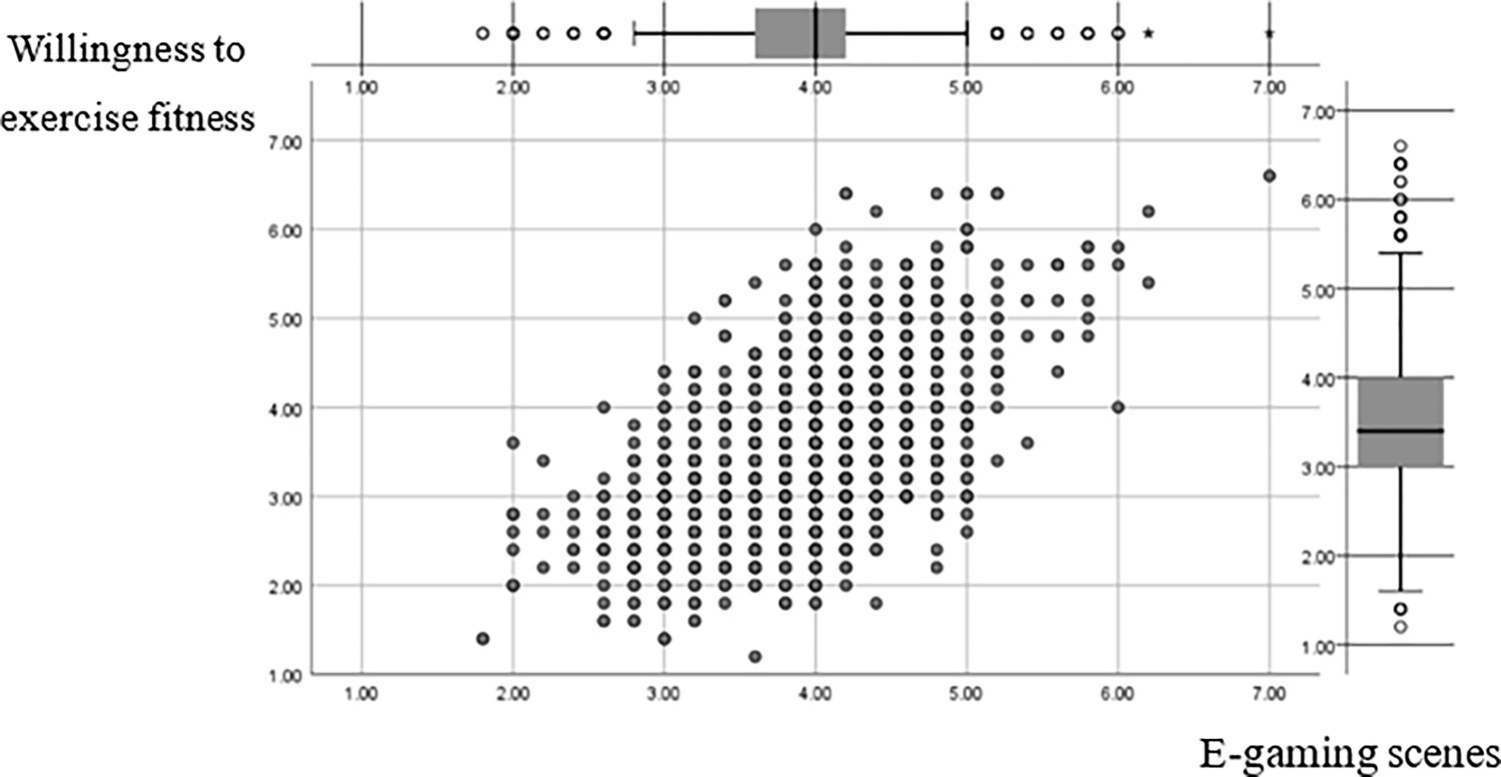
E-gaming scenes G-type graph of regression variables.

The goodness-of-fit test results are shown in Fig 2 based on the results of running the data in AMOS: CMIN/DF = 1.986 <3; GFI = 0.982, AGFI = = 0.976, RFI = 0.983, NFI = 0.985, TLI = 0.991, CFI = 0.993, the numerical results are greater than 0.9, indicating that the latent variables within the model and the fit between the indicator variables was satisfactory; RMR = 0.013<0.05, RMSEA = 0.025<0.05 judged that the structural equation model had a good fit and could reveal the influence of e-gaming experience on young people sports and fitness participation intention (Fig 1).

### 4.4. Testing hypotheses

#### 4.4.1. Test for direct effect

According to the test results in Table 3, the p-values of each direct effect path are <0.001, and the direct effects are significant; the direct effect values of e-gaming scene, virtual sports experience, and social presence perception on the intention to participate in sports and fitness are 0.399, 0.257, and 0.258, respectively, and the research hypotheses H_1_, H_3_, and H_6_ were valid; the direct effect values of e-gaming scene on virtual sports experience and social presence perception are 0.203 and 0.383, respectively, and the research hypotheses H_2_ and H_5_ were valid; the direct effect value of social presence perception on virtual sports experience is 0.301, and the research hypothesis H8 was valid. It can be seen that the hypotheses tested by the validation factor analysis in this study are all valid.

**Table 3:** Results of the path test.

Direct influence path	Non-normalized path coefficient	S.E.	C.R.	P-value	Normalized path coefficient
E-gaming scenes→ Virtual Sports Experience	0.107	0.016	6.815	***	0.203
Social Presence→ Virtual Sports Experience	0.243	0.026	9.444	***	0.301
E-gaming scenes→ Social Presence	0.251	0.018	13.981	***	0.383
E-gaming scenes→ Willingness to exercise fitness	0.268	0.017	16.169	***	0.399
Virtual Sports Experience→ Willingness to exercise fitness	0.327	0.033	9.769	***	0.257
Social Presence→ Willingness to exercise fitness	0.265	0.026	9.997	***	0.258

*P*<0.001(***).

#### 4.4.2. Test for mediation effect

Table 4 shows the results of the Bootstrap test: In the structural model of the effect of the e-gaming scene on the willingness to participate in sports and fitness, the direct effect and indirect effect are within the 95% confidence interval (excluding 0), and the p-value is <0.001, indicating that there are significant direct effects and indirect effects at the same time, and the model is a partial mediation model. In addition, "e-gaming scenario → virtual sports experience → willingness to participate in sports and fitness", "e-gaming scenario → social presence perception → willingness to participate in sports and fitness", "e-gaming scenario → social presence perception → virtual The three paths of "sports experience→ virtual sports experience→ voluntary sports and fitness participation" are within 95% confidence interval (excluding 0), and the p-value is <0.001, which verifies that the research hypotheses H4, H7, and H9 are all valid.

**Table 4:** Results of intermediate effect test.

Path of Influence	Effect Value	Boot SE	*P*	Bias-Corrected 95%CI		Percentile 95%CI	
				lower	Upper	lower	Upper
Total effect	0.580	0.019	0.001	0.544	0.616	0.543	0.616
Direct effect	0.399	0.023	0.001	0.354	0.445	0.354	0.445
Indirect effect	0.181	0.014	0.001	0.156	0.210	0.155	0.210
E-gaming scenes→ Virtual Sports Experience→ Willingness to exercise fitness	0.099	0.110	0.001	0.078	0.121	0.077	0.121
E-gaming scenes→ Social Presence→ Willingness to exercise fitness	0.052	0.009	0.001	0.035	0.071	0.035	0.070
E-gaming scenes→ Social Presence→ Virtual Sports Experience→ Willingness to exercise fitness	0.030	0.004	0.001	0.022	0.039	0.021	0.039

*10000 bootstrap samples.

## 5. Discussion and analysis

### 5.1. Analysis of direct effects

#### 5.1.1. Direct effect of e-gaming scenarios on the willingness to participate in sports and fitness

Sports video games, as part of the daily pastime and recreation of the general public, give different levels of stimulation to young people. Sociologist Bauman pointed out that today’s society is a "mobile society", and e-games have also started to "flow" in this "mobile society". From the fixed scenes of traditional games to the mobile scenes of cell phone online games, the boundaries of time and space are broken, and there is no need to consider the change in external scenes of reality. According to the scene theory, the game as a medium, from traditional games to cell phone online games, has changed drastically, which also brings changes to children’s lives. According to relevant studies, any form of physical activity is a typical process of mind-body interaction [46], and physical activity is a symbiotic movement that integrates body, mind, and spirit, which consists of three stages from shallow to deep: "physiological level of external stimulation, emotional level of interest feedback, and spiritual level of scenario integration" [47]. The physiological level is the logical starting point for young people participation, and the design of the game content, the portrayal of sports scenes, and the selection of background music will promote the willingness of young people to participate in sports and fitness. The emotional level is the internal factor for young people to choose sports and fitness participation, and the sense of achievement of reaching goals and the sense of participation in professional sports events will enhance young people’s willingness to participate again. As the driving force for young people to persist in real sports, the appeal of sports stars in the game, the tacit understanding of teamwork, and the competition of the game will enhance young people’s willingness to continue to participate. In view of this, game developers should: enrich the game design hierarchy and overall content playability; give the young people appropriate sensory stimulation to guide their interests; improve the consistency of the game with real sports images and data, establish a virtual community for game feedback; strengthen the game simulation and interactivity, and enrich the young people emotional experience. Secondly, the government and related social organizations should promote the construction of offline e-sports venues and carry out related e-sports events to promote the integration of online and offline scenarios, enhance the spiritual resonance and value identity between young people and sports, and promote young people participation in real sports (Fig 2).

#### 5.1.2. The direct effect of social presence perception on the willingness to participate in sports and fitness

**Fig 3:**
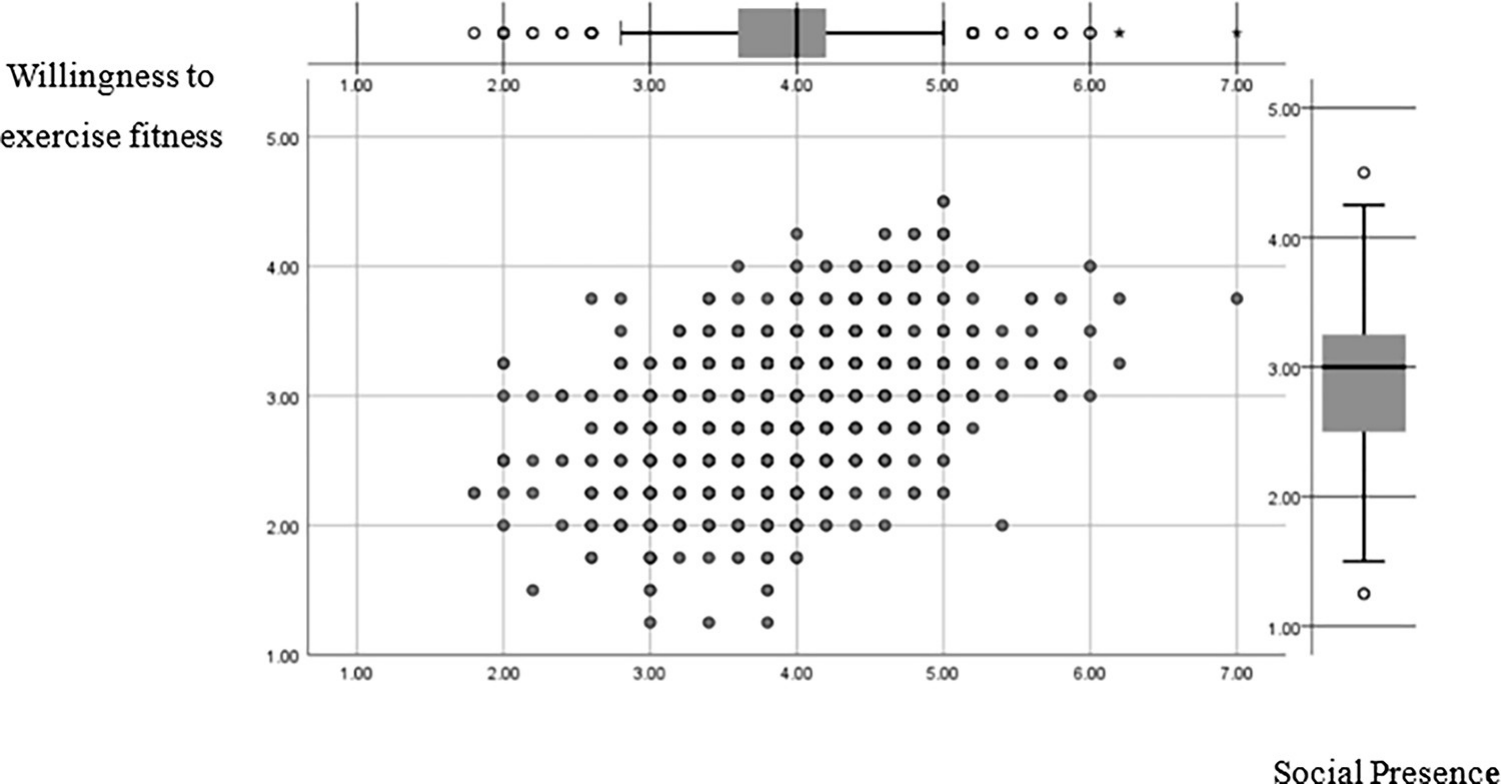
Social presence G-type graph of regression variables.

Different media will have different social presence due to differences in auditory, visual, and physical contact. In the area of communication technology and social research, the social presence hypothesis is a crucial theory. According to the social presence theory, we discovered that young people build social presence online that is comparable to interaction with others, such as social presence, immersion presence, and intimacy presence, due to the networkedness and interactivity of sports video games (Fig 3). Specifically: many NBA2K players hope to apply the basketball moves, skills, and fundamentals learned in the game to real-world play as a way to gain the envy and admiration of their peers and enhance their own sense of accomplishment and satisfaction [48,49]. Meanwhile, according to the trial-and-error psychological mechanism, when the narrative transmission about concepts, goals, or abilities provided within the game is vague, it leads to confusion among young people, which in turn leads to behaviors such as discussing with other players, checking game cheats, and trying to seek answers in the game and reality to confirm the rationality of their solutions [50]. Based on this, and based on the positive impact of sports video games in education, the attention to the narrative part of the game should be appropriately increased, focusing on the interactive narrative as well as other potential narrative forms; introducing strangeness mechanisms within the game, forcing young people to reflect on issues related to real sports while experiencing the game by adding cutting-edge information such as current affairs hotspots and technological information; meanwhile, providing young people with game process The visualization design and presentation allow young people to track their own behavior, choices, and learning progress through the visualization mode, which promotes young people’s social presence perception and effectively enhances their willingness to participate in sports and fitness.

#### 5.1.3. The direct effect of virtual sports experience on the willingness to participate in sports and fitness

**Fig 4:**
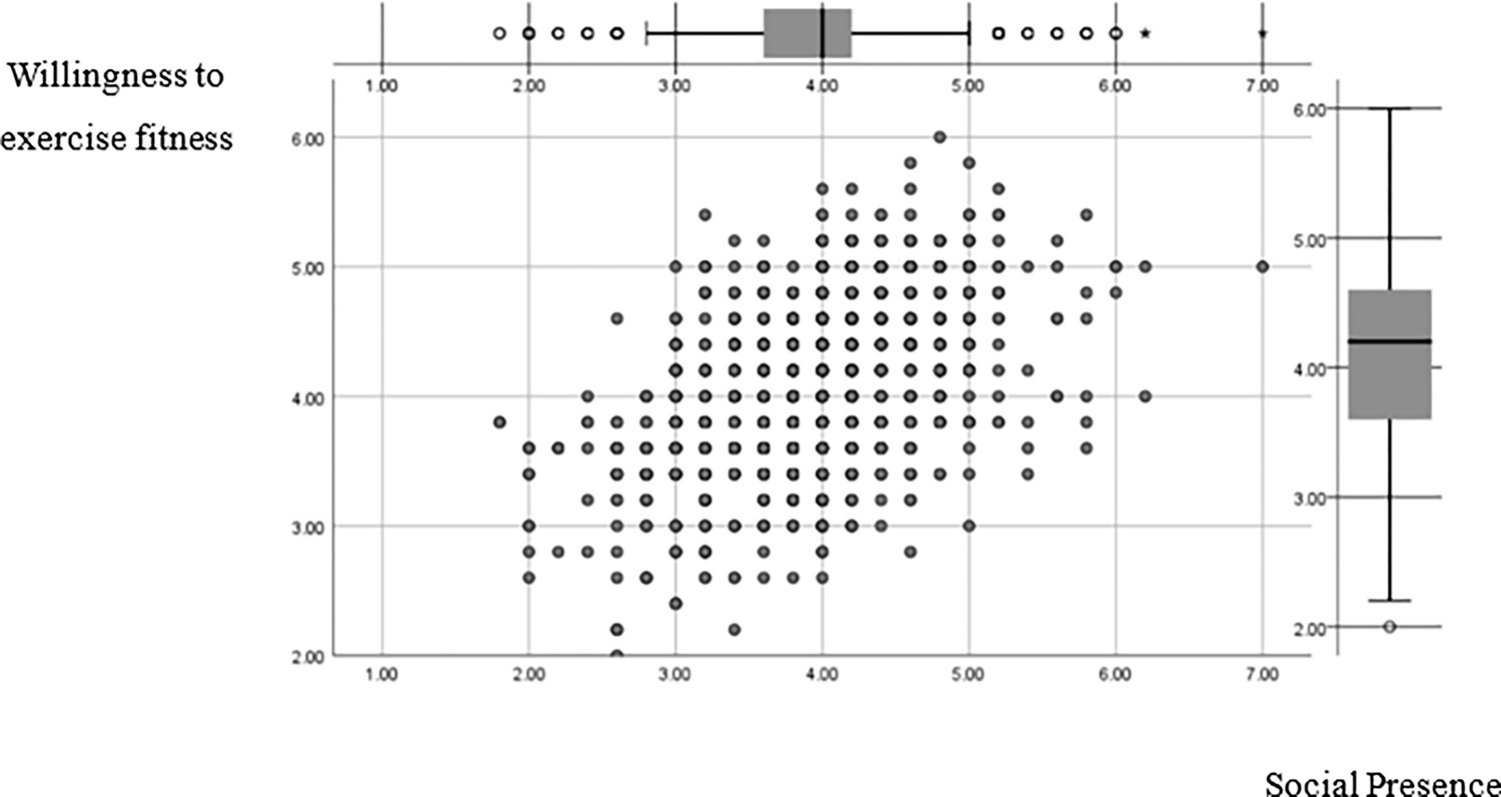
Social presence G-type graph of regression variables.

Virtual sports experience, as an important factor for young peoples’ understanding and awareness of sports activities, is the key to young peoples’ participation in sports. The gaming scenario experience, according to Choi and Kim (2004), is the key to video game behavior. They contend that in a balanced state of challenge and skill, players will have an optimal experience, enter a "state of immersion," and continue their gaming behavior. Studies have shown that young people with some sports experience easily combine virtual game experience with real sports and fitness participation experience. Young people generally participate in "sports" in their own style and manner, buying sports products related to sports stars or imitating them to show their love and support [51]. For example, they choose "Derrick Rose", "Messi", "Federer" and other sports superstars to play with, etc. At the same time, young people who have a high degree of virtual sports experience can use the game platform to shape and present themselves socially, to show their social status and enhance their social influence in real sports. In view of this, we should accurately grasp the trend of young people sports interests and enhance their cognitive experience and cognitive level by improving the authenticity of the game, strengthening the celebrity effect, and enhancing the fun teaching module. Simultaneously, increase young people access to game-related sports culture, knowledge, and skills thinking in order to motivate them to participate in realistic sports in a more scientific and reasonable manner, as well as to improve their physical and mental health and sports literacy (Fig 4).

Overall, the direct impact of e-gaming scenarios is substantial (β = 0.399, p0.01) and has a larger impact than both social presence and virtual sports experience (β = 0.257, p0.01) and willingness to participate in sports and fitness (β = 0.258, p0.01). It is clear that the scenario design of e-sports games has a significant role in luring the majority of young people to join and has a favorable effect on their propensity to engage in real-world sports. E-sports share traits with other competitive sports, most notably in their strong competitive and entertainment elements. E-sports have the impact of lowering physical and mental stress and enhancing free time since the virtual game scenario can provide participants with a strong sense of immersion. This is also in line with the results of this research, as reflected in the higher expectations of game players for the e-sports scene.

### 5.2. Analysis of the mediation effect

#### 5.2.1. Mediating effects of social proximity perception

Social presence theory suggests that the effectiveness of new media communication depends on the "immersive" state of perception that the medium provides to its users, and that the ability to interact with other participants in a shared space of meaning optimizes the effectiveness of their experience [52]. Social presence is an important factor influencing online interaction and collaboration in young people competitive gaming. Related studies have shown that the interactivity of sports video games affects users’ learning mechanisms and changes their habits in three ways: emotional, perceptual and behavioral. First, emotion, as the embodiment of the emotions generated by young people towards sports video games, is one of the important factors in changing young peoples’ attitudes towards sports [53]. The curiosity and the sense of accomplishment that young people feel when they reach their goals in the game trigger a shift in young peoples’ attitudes toward sports and drive them to engage or sustain their participation in sports. Secondly, perception is the process of young peoples’ processing of sport knowledge and information within sports video games and involves young peoples’ thinking and self-regulation within the game [54]. For example, tactical use as one of the key factors in winning a game [55], when young people observe game characters using blocking tactics to initiate an offense, they tend to apply them in the real game. Furthermore, behavior is the result of the combination of cognition and environment in virtual video games, and young peoples’ minds constantly flash to game actions such as basketball shooting, tennis swinging, soccer shooting, and boxing sparring, and imitate them in reality. In view of this, the emotional aspect should focus on the emotions and attitudes of young people, clarify the emotional changes and value demands of adolescents, enhance their interest in games and sports enthusiasm, and lay a solid cognitive foundation for establishing correct sports values. The perceptual aspect should be based on the psychological perception level of young people, strengthen the deep integration and interaction between young people and games, reasonably set game roles, tasks, and plots, and guide gamers to In terms of behavior, sports video games should be "design-based", based on the realistic needs of young people sports learning. Through detailed explanation of the technical actions of game characters, enriching the details of game content, strengthening human-computer interaction, etc., the game is designed to enhance the young people’s willingness to participate in sports and fitness.

#### 5.2.2. The mediating effect of virtual sports experience

The mediating effect of virtual sports experience is strong. The mediating effect of virtual sports experience is stronger than that of social field perception, and the reason for this is that virtual sports experience, as the most essential experience and psychological perception of sports for individuals, can influence young people’s cognition, thoughts or attitudes through sports knowledge, energy, culture and peripheral products, which is conducive to sports enthusiasts to link virtual game experience with real sports experience in order to achieve the physical level of "intuition" and "embodiment". According to the number of participants in e-sports games, we can simply divide video games into two categories: single-player and multi-player. In real sports and fitness life, many gamers have participated in both types of games, but, at the same time, tend to have a higher degree of preference for one of them. Specifically, sports video games contain a large number of realistic sports elements, such as in-game team logos, opening performances, and exciting replays designed to resemble the real event scene, and game characters are fully restored, which more realistically will show young people realistic sports scenes, deepen the level of young people virtual sports experience, mobilize young people sports participation enthusiasm, and attract young people to participate in realistic [56,57]. In view of this, sports video games should be based on improving the quality of young people’s visual, auditory and tactile experiences in all aspects, designing game modes with different difficulty levels, strengthening game scenario narratives, increasing the introduction of relevant sports culture background, and reducing the cognitive load of young people, so as to guide young people to actively participate in real sports.

#### 5.2.3. The chain mediation effect of social field perception and virtual sports experience

The mediating effect of social presence perception and virtual sports experience is weaker than the influence of independent paths, and is smaller than the direct influence of e-gaming scenarios on young peoples’ willingness to participate in sports and fitness. According to social exchange theory, whether an individual participates in resource exchange depends on the measurement of perceived benefits and perceived costs in the transaction process, which is implemented in the field of young people sports participation in terms of the degree of willingness to participate in real sports [58]. The reason for this is that the more complex the learning and cognitive content in the process of young peoples’ experience of sports video games, the more it reduces their motivation to participate in reality, which in turn affects their willingness to participate in sports and fitness [59]. In view of this, we should streamline the game selection interface, set reasonable game "archiving points", and create appropriate game "gap" periods, so that each young people can find a difficulty curve that suits him or her as much as possible. At the same time, detailed pictures and videos should be added to explain the difficult technical movements, so as to improve young peoples’ game experience and interest in sports, and to avoid the uncertainty of young peoples’ willingness to participate in sports and fitness to the maximum extent.

Overall, the mediating effect of e-gaming scenario on the willingness to participate in sports and fitness is more significant, with the total indirect effect size of OR 0.181,CI 0.156 0.210, p≤0.001, which shows that virtual sports experience and social presence perception play an important role in this process. The mediating effect of virtual sports experience was [OR] 0.099, 95%: CI 0.077–0.121, which was higher than that of social presence perception [OR] 0.052, 95%: CI 0.035–0.071. has a significant positive impact. Therefore, somatosensory games, as the top priority in the development of the video game industry, should promote the deep integration of modern technology and sports video games, fully apply somatosensory interaction technology to different sports scenarios, simulate the sense of fun and pleasure in real sports, fully mobilize the enthusiasm of young people to participate in sports, promote young people to improve sports knowledge and ability in a relaxed and pleasant game atmosphere, and gradually establish lifelong The aim is to inject new momentum to promote the efficient popularization of the national fitness strategy and the high-quality development of the sports, fitness, and leisure industries.

## 6. Conclusion

This paper explored the effects of e-gaming scenarios, social presence perceptions, and virtual sports experiences on young peoples’ willingness to participate in sports and fitness by constructing a structural equation model of the effects of e-gaming scenarios on young peoples’ willingness to participate in sports and fitness. The empirical tests revealed that the e-gaming scenario, virtual sports experience, and social presence perception all have direct effects on young peoples’ willingness to participate in sports and fitness. Among them, the direct effect value of e-gaming scenarios is the largest, and its features of entertainment, interactivity, and simulation can significantly influence young people’s willingness to participate in sports and fitness and attract them to participate in real sports. In the intermediate effect test, the largest intermediate effect value was found for virtual sports experience, indicating that sports video games enable young people to improve their virtual sports experience content, such as competitive skills and combat experience, in a simulated realistic environment and acquire the basic knowledge and skills necessary for real sports, which helps young people cultivate positive sports values and increase their willingness to participate in sports and fitness. Through a variety of social field perception strategies, young people continuously reflect on the sports phenomena in games and reality, form enthusiasm and curiosity about real sports, and solve the sports problems encountered in games and reality by consulting relevant sports knowledge, discussing with peers, and practicing, so as to promote young peoples’ participation in real sports. This study also has some limitations, chief among them being that (1) it is a cross-sectional investigation with a focus on questionnaires that participants administered themselves, and information gathered during the same time period may affect the mediating impact and bias the parameters, and (2) it focuses primarily on the personal traits and hands-on factors of young people gamers in the context of e-sports games while ignoring external pressure and influence. As a result, it is challenging to accurately control for the factors of game type and playing duration while conducting data analysis, and e-sports players’ abilities and game complexity are not taken into account. Future research needs to consider the above factors to further clarify the causal relationship between e-gaming experience and realistic willingness to participate and its underlying mechanisms.
